# DXA-derived visceral adipose tissue reference values and metabolic syndrome risk threshold in an Algerian adult population

**DOI:** 10.1371/journal.pone.0331867

**Published:** 2025-09-09

**Authors:** Mohammed Hadi Bestaoui, Ali Lounici, Amar Tebaibia, Latifa Henaoui, Nawal Brikci-Nigassa, Houssem Baghous, Amel Bensefia

**Affiliations:** 1 Internal Medicine Department, Tlemcen University Hospital, Tlemcen, Algeria; 2 Diabetes Research Laboratory, Faculty of Medicine, University of Abou Bekr Belkaid Tlemcen, Tlemcen, Algeria; 3 Internal Medicine Department, El Biar Hospital, Algiers, Algeria; 4 Epidemiology Department, Tlemcen University Hospital, Tlemcen, Algeria; 5 Biochemistry Department, Tlemcen University Hospital, Tlemcen, Algeria; 6 Diabetology and Endocrinology Department, Oran University Hospital, Oran, Algeria; Athens Medical Group, Psychiko Clinic, GREECE

## Abstract

**Background:**

Visceral adipose tissue (VAT) is associated with several cardiometabolic risk factors, particularly metabolic syndrome and insulin resistance. Reference values for VAT vary across populations, genders, and ages. Data on visceral fat in the Algerian population are lacking. This study aimed to establish reference values for VAT in a general adult population. The secondary objectives were to determine cardiometabolic consequences and to propose suggested threshold values for VAT to predict metabolic syndrome.

**Materials and methods:**

This cross-sectional, analytical study randomly selected participants from the electoral list of Tlemcen, Algeria. VAT was measured using dual-energy X-ray absorptiometry (DXA) General Electric Healthcare^©^ Lunar iDXA.

**Results:**

A total of 301 adults (147 men and 154 women) with a mean age of 49.3 ± 15.1 years participated. The median (25^th^-75^th^ percentiles) VAT mass was 1364 g (690–2049) in men and 1060 g (585–1590) in women. Binary logistic regression analyses demonstrated that cardiometabolic risk factors, including hypertension, type 2 diabetes, dyslipidemia, metabolic syndrome, insulin resistance according to HOMA2-IR, hepatic steatosis, and sleep apnea syndrome, were significantly dependent on VAT mass. Threshold values for VAT to predict metabolic syndrome (according to International Diabetes Federation) were ≥ 1369 g in men (sensitivity: 86.2%, specificity: 74.2%, Youden’s index: 0.604) and ≥ 1082 g in women (sensitivity: 76.3%, specificity: 76.9%, Youden’s index: 0.532).

**Conclusion:**

This study provides reference values for VAT in an urban Algerian adult population and highlights its importance in assessing cardiometabolic risk.

## Introduction

Obesity is a highly heterogeneous disease characterized by excess adipose tissue that causes negative health effects [1]. Among adults, its prevalence has more than doubled globally since 1990 [2]. The economic burden of obesity for society is also considerable, representing between 0.05% and 2.42% of a country’s gross domestic product [3]. In Algeria, the second STEPS-wise survey (2016–2017) conducted by the Ministry of Health with the support of the WHO revealed a much higher prevalence of obesity among adult women (30.1%) than men (14.1%) [4]. More recently, a multicenter observational study involving 3547 Algerian adults found a prevalence of overweight of 36% and obesity of 36.6% [5].

The current definition of obesity on the basis of body mass index (BMI) has several limitations. BMI does not differentiate between fat and lean mass or account for differences in body fat distribution [1].

Abdominal obesity is usually quantified using waist circumference, an essential measurement in clinical practice but one that does not distinguish between subcutaneous and visceral adipose tissue (VAT) [6]. Subcutaneous and visceral abdominal adipose tissues contribute differently to cardiometabolic risk, with excess visceral fat being more deleterious [7].

Classical anthropometric measures, such as BMI, waist circumference, waist-to-hip ratio, and waist-to-height ratio, are simple to use and show a strong positive correlation with VAT mass [8]. However, individuals with normal BMI values can present higher VAT accumulation, resulting in greater risk than estimated by conventional anthropometric measurements alone. Therefore, it is in the public interest to develop strategies for improved phenotyping, prevention, and treatment of obesity and related diseases.

Excess VAT promotes insulin resistance [9] and is associated with several chronic conditions, such as hypertension [10], dyslipidemia [11], type 2 diabetes [12,13], and metabolic syndrome [14]. It also promotes the development of cardiovascular diseases [15] and increases all-cause mortality [16].

Much research on VAT attempts to understand the mechanisms behind cardiometabolic diseases. This fat promotes ectopic lipid deposits, accumulating inside and around organs and thus impairing their functions. The liver is one of the major organs that undergoes this dysfunction, which is exacerbated by obesity. This leads to increased production of deleterious metabolites that, via the portal vein, directly disrupt hepatic metabolism, a central element of metabolic homeostasis [17]. Chronic low-grade inflammation, fibrosis, and hypoxia are major contributors to adipose tissue dysfunction [18].

Dual-energy X-ray absorptiometry (DXA) is one of the most commonly used methods to determine body composition. It allows for estimating bone mineral density, the quantity and distribution of fat mass, and lean mass. It is generally the preferred method in a clinical setting when a rigorous determination of body composition is required. A recent advancement in whole-body DXA is the ability to evaluate VAT. This can be achieved using General Electric (GE) Healthcare^©^ and Hologic^©^ devices, equipped with software such as CoreScan™ (GE-Lunar) and InnerCore™ (Hologic) [19].

This method uses the attenuation of X-rays from two energy sources to provide a three-compartment model of body composition (bone mass, fat mass, and lean mass), depending on the density and thickness of anatomical structures and tissues. VAT is calculated automatically by subtracting the subcutaneous adipose tissue from the android fat mass.

The main advantages of DXA over other VAT quantification methods are lower radiation exposure than computed tomography (CT), the radiation dose for patients undergoing DXA is considered negligible [20], and lower cost than magnetic resonance imaging (MRI).

VAT measured by DXA strongly correlated with VAT measured by CT (R² = 0.957) [21] and MRI (R² = 0.82 for females and R² = 0.86 for males) [22].

The main limitations of this method are the differences in measurement algorithms and acquisition areas between various DXA devices [23]. It also has limitations regarding its performance in very obese persons. However, half-scans in obese persons can provide an accurate analysis of body composition [24].

DXA-derived VAT reference values according to gender, age, and ethnicity are available for different populations worldwide [8,25–32]. However, there is a significant gap in data for the Maghreb region, as no reference values for VAT currently exist for the general population of Algeria or its neighbouring countries. This highlights a crucial need for research in this area to better understand and address health risks associated with VAT accumulation in this population.

This study aimed to establish reference values for VAT measured by GE Lunar iDXA in a general adult Algerian population. Secondary objectives were to determine cardiometabolic consequences and to propose suggested threshold values for VAT to predict metabolic syndrome.

## Materials and methods

### Study population

We conducted a cross-sectional observational study with an analytical aim over two and a half years, from March 13, 2022 to September 13, 2024, in a general urban adult population in Algeria. Participants were randomly selected from the electoral list of the municipality of Tlemcen, located in the northwest of Algeria, which includes 119 519 voters distributed across 36 centres and 327 voting offices. They were invited by mail to participate in the study.

We included participants aged 18–90 years who responded positively to the invitation. Exclusion criteria included individuals under 18 years and over 90 years, pregnant women, those weighing more than 227 kg (DXA table weight limit), bedridden individuals due to illness, and individuals with decompensated psychiatric disorders.

The required number of subjects for this study, calculated using the MedCalc software (v.22.021), was 142 men and 69 women. In total, we included 301 participants (147 men and 154 women). The detailed protocol available on protocols.io (DOI: dx.doi.org/10.17504/protocols.io.14egnrxxql5d/v1)

**Fig 1:**
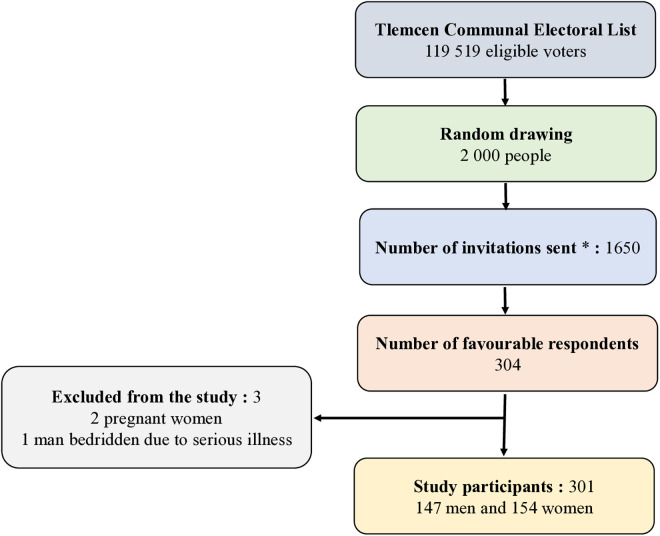
Study Flow Diagram. * Periodically and in order of drawing, 100 invitations were sent. A total of 1650 invitations were sent until the number of subjects required for the study was reached for both sexes.

The number of people invited, those who agreed to participate, and those included in the final analysis are shown in Fig 1.

### Ethical considerations

The study complied with all applicable institutional and governmental regulations regarding the ethical use of human volunteers and with the terms of the Declaration of Helsinki.

The scientific committee of Tlemcen University Hospital, via its Ethics Committee, approved the study protocol (approval number: 12-CSCHUT-2022), and all participants provided informed verbal consent after responding favourably to the invitation.

Verbal informed consent was chosen for this study due to the specific demographic characteristics of the local population. A portion of participants speak a local dialect, which may hinder their comprehension of both classical Arabic and French. Additionally, some individuals within this population may have low educational attainment, making written consent impractical.

The study’s procedures, objectives, and all examinations, including DXA scans, were thoroughly explained to participants. Particular emphasis was placed on the minimal radiation risk associated with DXA. These detailed explanations were also supported by a comprehensive invitation and information letter provided to each potential participant. Participants were explicitly informed that their involvement was entirely voluntary and that the study protocol posed no specific risks.

Verbal consent was attested on a dedicated consent form completed by the principal investigator. This form recorded the participant’s full name, date of birth, and date of consent approval. This documentation was then securely appended to the participant’s data collection form. The use of verbal consent formally approved by the aforementioned Ethics Committee.

### Study measures

#### Adiposity measures.

**Anthropometric measurements:** Weight and height were measured with light clothing and no shoes in kilograms (kg) and in centimetres (cm) using the OMRON HN 289 electronic scale and height chart.

Waist and hip circumference were measured in centimetres with measuring tape at the level of the umbilicus (halfway between the last rib and the upper iliac spine) and the greater trochanters, respectively.

BMI (weight in kg divided by height in meters (m) squared) was defined as underweight (<18.5 kg/m^2^), normal (18.5–24.9 kg/m^2^), overweight (25–29.9 kg/m^2^), or obese (≥ 30 kg/m^2^) with class I obesity (30–34.9 kg/m^2^), class II obesity (35–39.9 kg/m^2^) and class III obesity (≥40 kg/m^2^) [33]. The waist-hip ratio and waist-height ratio were calculated.

**DXA measurements:** Whole-body scans were conducted with a Lunar iDXA^TM^ (GE Healthcare^©^, USA) DXA scanner. Body composition parameters were analysed using the software enCORE^TM^ (version 18), and VAT was measured with the CoreScan^TM^ (GE Healthcare^©^, USA). Daily quality control was conducted, and calibration of the model was performed according to the manufacturer’s protocol. Participants were instructed to remove all metal objects, such as jewellery and watches. Shoes, jeans, and all clothes containing zippers or buttons, as well as bras with underwires, had to be removed.

VAT was expressed as a mass in grams (g), volume in cubic centimetres (cm^3^), and area in square centimetres (cm^2^). Other body composition parameters determined are:

- Subcutaneous adipose tissue mass (g)
- Total and regional fat mass (%)
- Fat mass index: fat mass/height^2^ (kg/m^2^)
- Appendicular lean mass index: lean mass of four limbs/height^2^ (kg/m^2^)
- Dual femur and anteroposterior spine bone mineral density (g/cm^2^)

All scans were performed and analysed by a single trained operator per a standard protocol provided by the manufacturer.

#### Cardiometabolic risk factors.

The cardiometabolic risk factors and other diseases associated with obesity that were assessed include: hypertension, type 2 diabetes and prediabetes, dyslipidemia, smoking, metabolic syndrome, chronic kidney disease, family history of cardiovascular events, cardiovascular events (coronary artery disease, stroke, peripheral arterial disease), sleep apnea syndrome, polycystic ovary syndrome, gallstones, osteoarthritis, hypothyroidism, hepatic steatosis, and history of cancers.

Blood pressure was measured three times, with the average of the last two readings taken using an electronic blood pressure monitor (OMRON model: M3 (HEM-7131-E)).

Blood samples were collected in the morning after a 12-hour fast. The analyses were performed at the laboratory of the Tlemcen University Hospital including glucose (g/l), urea (g/l), creatinine (mg/l) with the calculation of glomerular filtration rate (GFR) according to CKD-EPI, total cholesterol (g/l), HDL cholesterol (g/l), triglycerides (g/l), calculated LDL cholesterol (g/l), AST (IU/l), ALT (IU/l), uric acid (mg/l), complete blood count, glycated hemoglobin (A1c in %) by high-performance liquid chromatography, and C-peptide (ng/ml) by chemiluminescence.

Insulin resistance was assessed using the Homeostatic Model Assessment (HOMA2-IR) score with a threshold of 1.80 [34]. The other scores calculated were the triglycerides-glucose index, triglycerides HDL cholesterol ratio, Fibrosis-4 Index (FIB-4) [35], and the visceral adipose index (VAI) [36].

The criteria for metabolic syndrome applied were those of the International Diabetes Federation (IDF) harmonized in 2009 [37]: increased waist circumference ≥ 94 cm in men and ≥ 80 cm in women, elevated triglycerides ≥ 1.50 g/l (1.7 mmol/L) (or treatment for dyslipidemia), low HDL cholesterol < 0.40 g/l (1.03 mmol/L) in men and < 0.50 g/l (1.29 mmol/L) in women (or treatment for dyslipidemia), elevated fasting glucose ≥ 1 g/l (or treatment for type 2 diabetes), and elevated blood pressure: systolic ≥ 130 mm Hg and diastolic ≥ 85 mm Hg (or treatment for hypertension). Metabolic syndrome was defined as the presence of three or more of the components including waist circumference.

### Statistical analysis

The IBM SPSS Statistics 27.0 (IBM Corp., Armonk, New York, USA) software was used for statistical analyses. *p-*values were considered significant at the 0.05 level. VAT mass (g) and volume (cm^3^) were highly similar in all analyses; thus, VAT mass is presented in both bivariate and multivariate analyses.

For the descriptive analysis, the results were expressed as means ± standard deviations and/or medians (with 25^th^ and 75^th^ percentiles) for quantitative variables and as percentages for qualitative variables. The main comparisons were made based on gender and age groups.

After verifying normality (Shapiro-Wilk test) and equality of variances (Levene’s test), continuous quantitative variables were compared using the non-parametric Mann-Whitney and Kruskal-Wallis tests for variables that do not follow a normal distribution and the student’s t- test for variables that follow a normal distribution.

The Chi-square [χ2] test and Fisher’s exact test were used to compare qualitative variables.

VAT mass did not follow a normal distribution, as shown in supplementary materials (S1 Fig).

Non-parametric partial correlations by gender, adjusted for age, were performed between VAT mass and quantitative variables. Binary logistic regressions, using the enter method, were conducted to analyse the relationship between VAT and the main qualitative variables, which were considered dependent variables. VAT mass and age were included as independent variables.

A receiver operating characteristic (ROC) curve analysis of VAT mass in predicting metabolic syndrome was performed on both sexes to deduce sensitivity and specificity. Additionally, Youden’s index was calculated using the formula (sensitivity − (1 − specificity)) [38], to present suggested threshold values for VAT mass based on gender using estimated optimal cutoff values. The optimal threshold value of VAT for predicting metabolic syndrome corresponds to the highest Youden’s index.

## Results

The mean age was 45.7 ± 15.2 and 52.8 ± 14.2 years (range: 18.6 to 88.2 years), and the mean BMI was 26.9 ± 4.92 and 29.9 ± 6.51 kg/m^2^, in men and women, respectively.

27.2% of the population had a normal BMI, 35.9% were overweight, and 35.5% were obese (24.9% with class I obesity, 5% with class II obesity, and 5.6% with class III obesity).

The main cardiometabolic risk factors identified were hypertension in 35.9%, type 2 diabetes in 26.2%, prediabetes in 21.9%, dyslipidemia in 25.6%, active smoking in 11.0%, and metabolic syndrome (according to the IDF) in 44.5%.

Insulin resistance, assessed by a HOMA2-IR score greater than 1.8, was found in 58.6% of men and 67.6% of women, with no statistically significant difference between the genders (p = 0.114).

The general characteristics of the study population are represented in Table 1. Other general characteristics are detailed in the supplementary materials (S1 Table).

**Table 1:** General characteristics of the study population.

	All (N = 301)	Men (N = 147)	Women (N = 153)	p-value men vs. women
**Age** (years)	49.3 ± 15.1	45.7 ± 15,2	52.8 ± 14.2	< 0.001*
**Anthropometric parameters**				
Body Mass Index (kg/m^2^)	28.5 ± 5.97	26.9 ± 4.92	29.9 ± 6.51	< 0.001*
< 18.5	4 (1.3%)	3 (2%)	1 (0.6%)	< 0.001*
18.5–24.9	82 (27.2%)	50 (34%)	32 (20.8%)	
25–29.9	108 (35.9%)	55 (37.4%)	53 (34.4%)	
30–34.9	75 (24.9%)	33 (22.4%)	42 (27.3%)	
35–39.9	15 (5%)	2 (1.4%)	13 (8.4%)	
≥ 40	17 (5.6%)	4 (2.7%)	13 (8.4%)	
Waist circumference (cm)	97.1 ± 13.7	96.7 ± 12.2	97.5 ± 14.9	0.910
Hip circumference (cm)	102.1 ± 11.2	100.5 ± 8.09	103.5 ± 13,4	0.007*
Waist-to-hip ratio	0.948 ± 0.08	0.956 ± 0.07	0.940 ± 0.09	0.020*
Waist-to-height ratio	0.578 ± 0.09	0.545 ± 0.07	0.608 ± 0.09	< 0.001*
**Cardiometabolic risk factors**				
Hypertension	108 (35.9%)	44 (29.9%)	64 (41.6%)	0.036*
Type 2 diabetes	79 (26.2%)	34 (23.1%)	45 (29.2%)	0.230
Prediabetes	66 (21.9%)	33 (22.4%)	33 (21.4%)	0.831
Dyslipidemia	77 (25.6%)	38 (25.9%)	39 (25.3%)	0.917
Smoking	33 (11.0%)	33 (22.4%)	0	< 0.001*
Metabolic syndrome (IDF)	134 (44.5%)	58 (39.5%)	76 (49.4)	0.084
Chronic kidney disease	16 (5.3%)	7 (4.8%)	9 (5.8%)	0.676
Family history of cardiovascular events	24 (8.0%)	8 (5.4%)	16 (10.4%)	0.113
Hepatic steatosis	15 (5.0%)	10 (6.8%)	5 (3.2%)	0.156
Sleep apnea syndrome	14 (4.7%)	5 (3.4%)	9 (5.8%)	0.314
**Cardiovascular events**				
Coronary artery disease	6 (2.0%)	4 (2.7%)	2 (1.3%)	0.377
Stroke	6 (2.0%)	3 (2.0%)	3 (1.9%)	0.954
Peripheral arterial disease	1 (0.3%)	1 (0.7%)	0	0.305
**Others diseases**				
Polycystic ovary syndrome	–	–	8 (5.2%)	–
Gallstones	35 (11.6%)	8 (5.4%)	27 (17.5%)	0.001*
Osteoarthritis	88 (29.2%)	17 (11.6%)	71 (46.1%)	< 0.001*
Hypothyroidism	50 (16.6%)	9 (6.1%)	41 (26.6%)	< 0.001*
History of cancers	6 (2.0%)	1 (0.7%)	5 (3.2%)	0.111
**Healthy person** ^a^	124 (41.2%)	66 (44.9%)	58 (37.7%)	0.202
**Blood pressure**				
Systolic blood pressure (mm Hg)	126.65 ± 16.7	127.01 ± 15.3	126.31 ± 17.9	0.442
Diastolic blood pressure (mm Hg)	79.06 ± 9.24	79.91 ± 9.49	78.24 ± 8.95	0.117
**Blood tests**				
Glucose (g/l)	1.08 ± 0.39	1.06 ± 0.38	1.11 ± 0.40	0.175
C-peptide ^b^ (ng/ml)	3.40 ± 2.07	3.33 ± 2.17	3.47 ± 1.97	0.249
A1c (%)	5.92 ± 1.37	5.71 ± 1.13	6.13 ± 1.53	0.011*
Total cholesterol (g/l)	1.73 ± 0.39	1.67 ± 0.39	1.78 ± 0.39	0.021*
HDL cholesterol (g/l)	0.46 ± 0.13	0.41 ± 0.11	0.50 ± 0.13	< 0.001*
LDL cholesterol (g/l)	1.02 ± 0.34	0.99 ± 0.32	1.06 ± 0.35	0.151
Triglycerides (g/l)	1.18 ± 0.63	1.27 ± 0.68	1.10 ± 0.57	0.082
Non-HDL cholesterol (g/l)	1.26 ± 0.36	1.26 ± 0.36	1.27 ± 0.37	0.767
Creatinemia (mg/l)	8.40 ± 1.92	9.45 ± 1.53	7.40 ± 1.71	< 0.001*
AST (IU/l)	24.6 ± 10.1	25.7 ± 9.41	23.6 ± 10.8	0.005*
ALT (IU/l)	25.3 ± 16.3	28.5 ± 17.0	22.2 ± 15.1	< 0.001*
Uric acid ^b^ (mg/l)	49.4 ± 14.2	56.9 ± 13.4	44.1 ± 12.4	< 0.001*
**Scores**				
HOMA 2-IR ^b^	2.72 ± 2.08	2.74 ± 2.53	2.70 ± 1.54	0.184
Triglycerides -Glucose index	8.61 ± 0.60	8.65 ± 0.62	8.57 ± 0.58	0.441
Triglycerides HDL cholesterol ratio	2.93 ± 2.23	3.45 ± 2.49	2.43 ± 1.83	< 0.001*
FIB-4	1.05 ± 0.58	1.00 ± 0.61	1.10 ± 0.55	0.024*
VAI	2.05 ± 1.55	1.99 ± 1.46	2.10 ± 1.64	0.203

The results are expressed as mean ± standard deviation for quantitative variables. ALT: Alanine Aminotransferase, AST: Aspartate Aminotransferase, HOMA2-IR: Homeostasis Model Assessment 2 Insulin Resistance, IDF: International Diabetes Federation, VAI: Visceral Adipose Index. *: significant difference between men and women with p-value < 0.05. ^a^: An individual is considered healthy if they are free from conditions like hypertension, type 2 diabetes, dyslipidemia, metabolic syndrome, cardiovascular events, sleep apnea syndrome, and hepatic steatosis. ^b^: Parameters analysed in: 291 participants for C-peptide, 288 participants for HOMA2-IR and 236 participants for uric acid.

### VAT reference values

VAT (mass, volume, and area) and other DXA-derived body composition variables by gender and age group are represented in Table 2.

**Table 2:** Visceral adipose tissue and other DXA-derived body composition variables by gender and age groups.

Age (years)							
	All	18 to ≤ 30	> 30 to ≤ 40	> 40 to ≤ 50	>50 to ≤ 60	> 60 to ≤ 70	> 70–88
**Men**							
**N**	147	26	26	40	30	15	10
**VAT mass** (g), median	1364* (690 −2049)	366 (174-629)	1121* (771-1529)	1730* (1199-2220)	1555 (996-2059)	2081* (1348-2541)	1845 (1377-2078)
mean ± SD	1446* ± 893	438 ± 341	1366* ± 907	1782* ± 828	1568 ± 745	1991* ± 807	1745 ± 690
**VAT volume** (cm^3^)	1446* (731-2172)	388 (184-667)	1188* (818-1621)	1833* (1272-2354)	1648 (1056-2182)	2206* (1429-2694)	1955 (1460-2202)
**VAT area** (cm^2^)	154 (79-235)	43* (20-69)	133* (85-185)	181* (130-247)	179 (114-233)	232* (150-290)	210 (159-249)
**SAT mass** (g)	1265* (916-1716)	907* (489-1664)	1297* (980-1875)	1650 (1108-2441)	1160* (853-1519)	1150* (886-1431)	1192 (828-1464)
**Total FM** (%)	32.4* (28.9-36.7)	27.0* (20.0-31.7)	30.7* (28.7-34.9)	35.2* (30.1-39.4)	32.3* (29.3-34.5)	34.5* (32.3-36.7)	35.1* (31.4-36.8)
**Android FM** (%)	39.8* (32.6-45.6)	27,4* (19.4-36.5)	37.3* (32.6-43.3)	45.5* (37.1-50.2)	41.3* (34.1-44.7)	43.8* (37.3-47.7)	42.3* (38.7-45)
**Gynoid FM** (%)	32.3* (28.7-36.9)	30.2* (20.6-36)	32* (29-35.8)	34.9* (29.6-39.3)	30.8* (28.5-33.9)	32.5* (30.2-35.9)	35.2* (28.5-36.6)
**Trunk FM** (%)	36.8* (30.7-41.7)	26.9* (20.2-35)	34.6* (30.6-40.3)	41.6* (34.1-46.2)	38.2* (32.1-40.5)	39.9* (36.5-44)	39.9* (35.7-40.9)
**FMI** (kg/m^2^)	8.1* (6.6-10.4)	5.6* (3.7-7.9)	7.6* (6.5-10.2)	9.3* (7.3-12.4)	8.3* (6.7-9.4)	8.4* (8.0-10.3)	9.5 (6.8-10.3)
**ALMI** (kg/m^2^)	8.02* (7.35-8.83)	7.69* (7.04-8.77)	8.38* (7.51-8.90)	8.63* (7.85-9.27)	7.88* (7.37-8.62)	7.66* (6.88-8.30)	7.09 (6.84-8.21)
**Dual femur BMD** (g/cm^2^)	1.047* ± 0.147	1.077 ± 0.125	1.088* ± 0.178	1.056 ± 0.114	1.025* ± 0.145	1.023* ± 0.146	0.933* ± 0.192
**AP spine BMD** (g/cm^2^)	1.158* ± 0.168	1.151 ± 0.125	1.178 ± 0.150	1.141 ± 0.123	1.133* ± 0.207	1.198* ± 0.223	1.212* ± 0.245
**Women**							
**N**	153	10	21	33	31	45	14
**VAT mass** (g), median	1060* (585-1590)	567 (364-655)	667* (455-1113)	959* (663-1518)	1262 (582-1844)	1198* (801-1688)	1253 (727-2322)
mean ± SD	1144* ± 697	597 ± 329	754* ± 415	1056* ± 578	1329 ± 801	1295* ± 706	1431 ± 803
**VAT volume** (cm^3^)	1123* (620-1685)	601 (386-695)	707* (483-1180)	1017* (703-1608)	1338 (617-1955)	1270* (849-1789)	1327 (771-2462)
**VAT area** (cm^2^)	131 (72 −192)	67* (48-82)	77* (54-130)	113* (81-178)	145 (72-221)	151* (100-210)	168 (102-306)
**SAT mass** (g)	1804* (1276-2505)	1760* (1258-2070)	2300* (1519-2537)	1934 (1434-2583)	1712* (1218-2901)	1643* (1258-2619)	1503 (1046-2046)
**Total FM** (%)	45.0* (41.8-48.9)	43.0* (41.8-45.8)	44.8* (41.5-47.4)	44.8* (42.6-49.2)	43.3* (40.7-49.0)	46.0* (41.9-49.8)	45.2* (39.6-49.0)
**Android FM** (%)	49.7* (43.2-54.5)	44.4* (42.1-47.2)	48.5* (41.7-53.8)	50.5* (43.2-53.6)	49.1* (42.2-55.3)	51.7* (44.1-56)	51.2* (43.5-56.3)
**Gynoid FM** (%)	48.2* (44-51.7)	49.1* (45.4-52)	50.6* (47.9-53.8)	48.4* (47.1-51)	47.9* (42.5-51.7)	48.1* (42.2-51.8)	43.9* (41-49.3)
**Trunk FM** (%)	47* (42-51.7)	41.9* (41.4-45.9)	46.2* (41.3-50.6)	47.9* (42.4-50.6)	46.3* (41.1-52.2)	48.8* (43.5-53.5)	46.8* (41.7-52.2)
**FMI** (kg/m^2^)	12.5* (10.1-15.6)	10.8* (9.4-12.8)	12.9* (10.4-15.1)	13.1* (10.9-16.2)	11.7* (10.1-16.3)	13.0* (10.5-15.9)	12.9 (9.4-15.2)
**ALMI** (kg/m^2^)	6.83* (6.04-7.84)	6.54* (5.55-7.31)	6.82* (6.19-7.89)	7.01* (6.53-8.24)	6.84* (6.04-8.13)	6.69* (5.92-7.73)	6.68 (5.73-7.87)
**Dual femur BMD** (g/cm^2^)	0.934* ± 0.159	1.080 ± 0.154	0.989* ± 0.125	1.031 ± 0.155	0.904* ± 0.097	0.890* ± 0.136	0.732* ± 0.117
**AP spine BMD** (g/cm^2^)	1.044* ± 0.193	1.226 ± 0.120	1.180 ± 0.113	1.157 ± 0.159	1.015* ± 0.144	0.936* ± 0.171	0.857* ± 0.182

Normally distributed variables are represented by mean ± standard deviation. Non-normally distributed variables represented with median (25th percentile–75th percentile).

ALMI: Appendicular Lean Mass Index, AP: anteroposterior, BMD: Bone Mineral Density, FM: Fat Mass, FMI: Fat Mass Index, SAT: Subcutaneous Adipose Tissue, VAT: Visceral Adipose Tissue. *: significant difference between men and women with p-value < 0.05.

The VAT mass was higher in men than in women (median (25^th^–75^th^ percentiles): 1364 g (690–2049) vs. 1060 g (585–1590), p = 0.005). This difference was observed across different age groups, except for the 18–30 years range, where the VAT mass was higher in women, although not significantly (p = 0.08). VAT mass increased with age up to 70 years in men and up to 60 years in women.

Moreover, the subcutaneous adipose tissue mass was higher in women than in men, increasing and reaching its peak in the 30–40 years age group for women and in the 40–50 years age group for men. Total fat mass, android fat mass, gynoid fat mass, trunk fat mass, and fat mass index were also higher in women than in men, in contrast to appendicular lean mass index and bone mineral density.

VAT values percentiles by gender are detailed in the supplementary materials (S2 Table).

VAT mass by gender, age groups and health status are represented in the supplementary materials (S3 Table).

### Risk factors and cardiometabolic consequences of VAT

Non-parametric partial correlations by gender, adjusted for age, were performed between VAT mass and quantitative variables, and the results are represented in Table 3.

**Table 3:** Non-parametric partial correlations by gender adjusted for age between VAT mass and quantitative variables.

Quantitative variable	Correlation coefficient « r »		
	Men	Women	All
**Anthropometric parameters**			
BMI (kg/m^2^)	0.805 ^c^	0.827 ^c^	0.699 ^c^
Waist circumference (cm)	0.851 ^c^	0.848 ^c^	0.823 ^c^
Hip circumference (cm)	0.731 ^c^	0.751 ^c^	0.642 ^c^
Waist-to-hip ratio	0.709 ^c^	0.440 ^c^	0.594 ^c^
Waist-to-height ratio	0.799 ^c^	0.814 ^c^	0.649 ^c^
**Blood pressure**			
Systolic blood pressure (mm Hg)	0.106	0.270 ^c^	0.211 ^c^
Diastolic blood pressure (mm Hg)	0.325 ^c^	0.272 ^c^	0.340 ^c^
**DXA measurements**			
FMI (kg/m^2^)	0.867 ^c^	0.832 ^c^	0.535 ^c^
SAT mass (g)	0.770 ^c^	0.757 ^c^	0.607 ^c^
Total FM (%)	0.847 ^c^	0.724 ^c^	0.315 ^c^
Android FM (%)	0.911 ^c^	0.842 ^c^	0.646 ^c^
Gynoid FM (%)	0.664 ^c^	0.447 ^c^	0.096
Trunk FM (%)	0.913 ^c^	0.826 ^c^	0.559 ^c^
Arms FM (%)	0.641 ^c^	0.580 ^c^	0.091
Legs FM (%)	0.543 ^c^	0.242 ^b^	−0.015
Android to gynoid FM ratio	0.716 ^c^	0.752 ^c^	0.766 ^c^
Trunk to total FM ratio	0.725 ^c^	0.685 ^c^	0.756 ^c^
Legs to total FM ratio	−0.603 ^c^	−0.565 ^c^	−0.636 ^c^
ALMI (kg/m^2^)	0.587 ^c^	0.718 ^c^	0.688 ^c^
Dual femur BMD (g/cm^2^)	0.187 ^a^	0.468 ^c^	0.395 ^c^
**AP spine BMD** (g/cm^2^)	0.149	0.322 ^c^	0.296 ^c^
**Biological parameters**			
Glucose (g/l)	0.287 ^c^	0.418 ^c^	0.340 ^c^
C-Peptide (ng/ml)	0.467 ^c^	0.449 ^c^	0.442 ^c^
A1c (%)	0.353 ^c^	0.323 ^c^	0.318 ^c^
Creatinemia (mg/l)	0.159	0.073	0.250 ^c^
GFR (ml/min/1.73m^2^)	−0.216 ^b^	−0.110	−0.196 ^c^
Total cholesterol (g/l)	0.080	−0.067	−0.040
HDL cholesterol (g/l)	−0.300 ^c^	−0.349 ^c^	−0.415 ^c^
LDL cholesterol (g/l)	0.135	−0.037	0.033
Triglycerides (g/l)	0.381 ^c^	0.386 ^c^	0.410 ^c^
Non-HDL cholesterol (g/l)	0.159	0.059	0.104
Triglycerides HDLc ratio	0.419 ^c^	0.455 ^c^	0.495 ^c^
ALT (IU/l)	0.419 ^c^	0.317 ^c^	0.410 ^c^
AST (IU/l)	0.274 ^c^	0.099	0.219 ^c^
Uric acid (mg/l)	0.320 ^c^	0.401 ^c^	0.465 ^c^
**Scores**			
HOMA2-IR	0.467 ^c^	0.499 ^c^	0.468 ^c^
FIB-4	0.070	−0.085	0.013
Triglycerides – Glucose index	0.422 ^c^	0.462 ^c^	0.459 ^c^
VAI	0.425 ^c^	0.473 ^c^	0.443 ^c^

A1c: Glycated hemoglobin, ALT: Alanine Aminotransferase, AST: Aspartate Aminotransferase, ALMI: Appendicular Lean Mass Index, BMD: Bone Mineral Density, BMI: Body Mass Index, FIB-4: Fibrosis-4 Index, HOMA2-IR: Homeostasis Model Assessment 2 Insulin Resistance, FM: Fat mass, FMI: Fat Mass Index, GFR: Glomerular Filtration Rate, SAT: Subcutaneous Adipose Tissue, VAI: Visceral Adipose Index, VAT: Visceral Adipose Tissue. ^a^: p < 0.05, ^b^: p < 0.01, ^c^: p < 0.001.

Significant positive correlations were found with various anthropometric measurements (strongest with waist circumference, BMI, waist-to-height ratio, and hip circumference) and with different body composition measurements (strongest with trunk fat mass, android fat mass, fat mass index, total fat mass, trunk fat mass/total fat mass ratio, and android fat mass/gynoid fat mass ratio).

Significant positive correlations were found with C-peptide, the triglyceride-HDL cholesterol ratio, triglycerides, uric acid, ALT, A1c, and fasting glucose. HDL cholesterol and GFR showed a negative correlation. No correlation was found with LDL cholesterol, total cholesterol, or non-HDL cholesterol. Significant positive correlations were found with insulin resistance scores, with the strongest associations observed with the HOMA2-IR index, the VAI, and the triglyceride-glucose index.

Binary logistic regression analyses with VAT mass and age as independent variables demonstrated that cardiometabolic risk factors, including hypertension, type 2 diabetes, dyslipidemia, metabolic syndrome, insulin resistance according to HOMA2-IR, hepatic steatosis, and sleep apnea syndrome, were significantly dependent on VAT mass (Table 4).

**Table 4:** Binary logistic regression analyses with VAT mass, age, and cardiometabolic risk factors.

	B	Standard Error	Wald	p-value	Odds Ratio (95% CI)	R^2^
**Hypertension**						0.417
VAT Mass	0.0007	0.0002	13.43	<0.001*	1.0007 (1.0003 − 1.0010)	
Age	0.0955	0.0132	52.63	<0.001*	1.1002 (1.0722–1.1289)	
Constant	−6.500	0.787	68.24	<0.001*	0.002	
**Type 2 diabetes**						0.257
VAT Mass	0.0007	0.0002	17.10	<0.001*	1.0007 (1.0004–1.00011)	
Age	0.0552	0.0113	23.89	<0.001*	1.0568 (1.0336–1.0804)	
Constant	−5.017	0.689	53.08	<0.001*	0.007	
**Dyslipidemia**						0.213
VAT Mass	0.0008	0.0002	20.44	<0.001*	1.0008 (1.0005 - 1.0012)	
Age	0.0388	0.0107	13.04	<0.001*	1.0396 (1.0179 - 1.0617)	
Constant	−4.265	0.638	44.74	<0.001*	0.014	
**Metabolic syndrome** (IDF)						0.441
VAT Mass	0.0019	0.0002	57.71	<0.001*	1.0019 (1.0014 - 1.0024)	
Age	0.0257	0.0106	5.85	0.016*	1.0260 (1.0049–1.0476)	
Constant	−3.940	0.602	42.80	<0.001*	0.019	
**Insulin resistance by HOMA2-IR**						0.228
VAT Mass	0.0013	0.0002	33.43	<0.001*	1.0013 (1.0009 - 1.0018)	
Age	0.0011	0.0094	0.013	0.910	1.0011 (0.9828 − 1.0196)	
Constant	−1.053	0.448	5.535	0.019*	0.349	
**Hepatic steatosis**						0.168
VAT Mass	0.0012	0.0003	15.91	<0.001*	1.0012 (1.0006 - 1.0019)	
Age	−0.0045	0.0214	0.044	0.834	0.9955 (0.9547 - 1.0381)	
Constant	−4.812	1.240	15.05	<0.001*	0.008	
**Sleep apnea syndrome**						0.142
VAT Mass	0.0010	0.0003	9.65	0.002*	1.0010 (1.0004 - 1.0016)	
Age	0.0301	0.0212	2.011	0.156	1.0305 (0.9886–1.0742)	
Constant	−6.305	1.389	20.60	<0.001*	0.002	
**Chronic kidney disease**						0.270
VAT Mass	0.0004	0.0003	1.31	0.253	1.0004 (0.9997–1.0011)	
Age	0.1047	0.0234	19.05	<0.001*	1.1104 (1.0594–1.1639)	
Constant	−9.656	1.722	31.44	<0.001*	0.0001	

CI: Confidence Interval, HOMA2-IR: Homeostasis Model Assessment 2 Insulin Resistance, IDF: International Diabetes Federation, VAT: Visceral Adipose Tissue. *: significant difference with p-value < 0.05.

### Threshold values of VAT for metabolic syndrome

**Fig 2:**
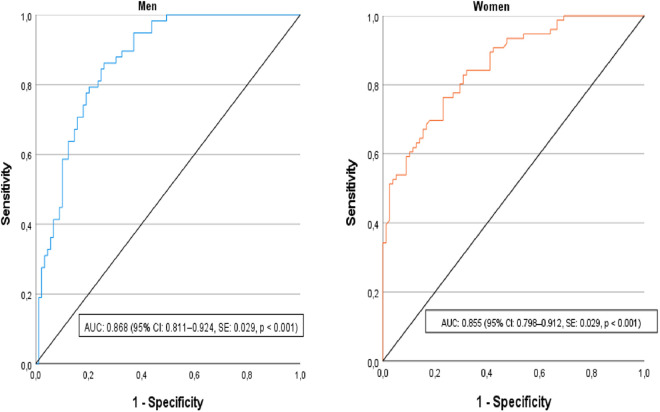
Receiver operating characteristics of VAT mass for identifying metabolic syndrome according to the IDF criteria in men and women. AUC: Area Under the Curve.

The analysis of ROC curves for VAT mass in predicting metabolic syndrome according to the IDF criteria showed good areas under the curve in both sexes: 0.868 (95% CI: 0.811–0.924, SE: 0.029, p < 0.001) for men and 0.855 (95% CI: 0.798–0.912, SE: 0.029, p < 0.001) for women (Fig 2).

Using Youden’s index, the threshold values for VAT mass were 1369 g (sensitivity: 86.2% and specificity: 74.2%) for men and 1082 g (sensitivity: 76.3% and specificity: 76.9%) for women (Table 5).

**Table 5:** Suggested threshold values of VAT derived from Youden’s index for metabolic syndrome.

	AUC (95% CI)	VAT thresholds values		Youden’s Index	Sensitivity (%)	Specificity (%)
		Mass (g)	Volume (cm^3^)			
**Men**	0.868 (0.811–0.924)	≥ 1369	≥ 1451	0.604	86.2	74.2
**Women**	0.855 (0.798–0.912)	≥ 1082	≥ 1147.5	0.532	76.3	76.9

AUC: Area Under the Curve, CI: Confidence Interval, VAT: Visceral adipose tissue.

Others suggested threshold values of VAT derived from Youden’s index for cardiometabolic risk factors (hypertension, type 2 diabetes, dyslipidemia, Insulin resistance by HOMA2-IR, hepatic steatosis and sleep apnea syndrome) are detailed in the supplementary materials (S4 Table).

## Discussion

This first cross-sectional Algerian study provided reference values for VAT measured by DXA (GE Lunar iDXA) in an urban adult population aged 18–88 years selected by random sampling. The practical implication of these results is the identification of at-risk individuals and the provision of management strategies based on the proposed threshold values for predicting metabolic syndrome.

DXA machines employ different algorithms for measuring VAT and utilise varying scan regions. GE Healthcare systems use a larger android scan region compared to Hologic systems, which employ a 5-cm region to estimate VAT at the L4/L5 spinal level, leading to inherent differences in the measured VAT. These discrepancies in methodology necessitate that measurements obtained from different systems cannot be directly interchanged. Recently, cross-calibration equations have been developed for VAT, subcutaneous adipose tissue, and total adipose tissue between GE Healthcare and Hologic DXA systems [23].

MRI and CT are considered the gold standard techniques for evaluation of VAT. A key difference is that DXA measures the overall chemical compartment of triglycerides, representing the body’s total fat mass. In contrast, CT and MRI quantify adipose tissue within a specific anatomical compartment. This anatomical compartment includes not only adipocytes but also other components like collagen, fibroblasts, and capillaries. Consequently, fat mass values obtained from DXA are typically slightly lower than those derived from cross-sectional imaging techniques like CT and MRI because DXA is specifically targeting triglycerides, whereas CT and MRI are measuring a broader tissue volume that includes non-fat components [39].

However, some constraints limit the widespread use of CT and MRI in clinical practice such as cost, the need for qualified professionals to perform and analyse these examinations, and significant irradiation (higher radiation dose delivered by CT compared to DXA) [40].

Analysis of the general characteristics of the study population reveals that the average age of women is higher than that of men. This could partly explain a higher BMI, A1c, and a higher frequency of hypertension. However, no significant difference in health status was observed between men and women. These differences may also be influenced by the cultural context, potentially contributing to variations in lifestyle and professional engagement observed between genders in Algeria. Additionally, hormonal changes during menopause often lead to an increase in VAT and an increased risk of obesity [41]. Other diseases such as hypothyroidism [42], osteoarthritis [43], and gallstones [44] are known to have a higher prevalence in women than in men and to increase with age.

VAT accumulation, a significant risk factor for metabolic diseases, is influenced by the complex interplay of age, gender, genetics, and ethnicity [45–51]. Specific mechanisms contributing to increased visceral fat storage during positive energy balance include sex hormones, local cortisol production, endocannabinoids, growth hormone, and dietary fructose [45]. Notably, men tend to accumulate more VAT with increasing total body fat, whereas in women, VAT volume is less impacted by overall body fat, highlighting a distinct sex dimorphism in fat patterning, largely attributed to sex hormones [45,51]. As individuals age, VAT generally increases due to fat redistribution and declining sex hormone levels, leading to dysfunctional adipose tissue and a low-grade inflammatory state [45,47]. Furthermore, ethnic differences are pronounced, with Asian populations often exhibiting higher VAT at lower BMIs compared to Europeans, while individuals of African ancestry tend to have less VAT at similar BMIs, underscoring the strong genetic component in these variations [45,46,50].

Age and sex-specific DXA-derived VAT reference values have been reported for different populations in Europe (Norway [27], Austria [25], Poland [52], Italy [30], and the United Kingdom [26]), the Middle East (Qatar [29] and Kuwait [28]), Oceania (Australia [8] and New Zealand [32]), and the United States of America [31].

In our study, we observed that the VAT mass was higher in men than in women and increased with age. Women exhibited a higher VAT mass compared to women in the previously mentioned populations. These differences may be attributable to significant variations in ethnicity, culture, number of pregnancies, physical activity, and dietary habits. Our study included a general population comprised of both healthy individuals and those with diseases, in contrast to some studies that only considered healthy subjects under 66 years [8,26,52]. In men, the most important increase in VAT mass is observed between the 18–30 years and 30–40 years age groups, while in women, it occurs between the 40–50 years and 50–60 years age groups.

Our results are consistent with several studies that also confirm the role of VAT in hypertension [53], type 2 diabetes [12], dyslipidemia and metabolic syndrome [27], hepatic steatosis [54], sleep apnea syndrome [55], and insulin resistance [56].

Significant heterogeneity exists in VAT threshold values for metabolic syndrome prediction, influenced by factors such as geographic location, age, imaging technique, metabolic profile, diagnostic definition, and recruitment methodology [14].

The presented reference values and threshold are applicable only to similar populations and measurements obtained using comparable equipment. Further research is required to validate these thresholds in our population.

### Strengths and limitations

This study provides the first Algerian, and more broadly, Maghrebian data on VAT assessed by GE LUNAR iDXA in a general adult population. Despite a random sampling of the population based on the electoral list, participation in the study remained voluntary upon invitation. Individuals more concerned about their health status, with existing pathologies, more obese, or simply older and with more free time were more likely to participate. Certain population categories, particularly younger individuals, may therefore be underrepresented.

Our study is monocentric and focused on an urban adult population. Generalizing the results to the entire Algerian population with its diverse socioeconomic, cultural, and environmental factors should be done with caution. Further studies are needed. Although the analysis was conducted based on gender and age groups, with correlations adjusted for age, other potential confounding factors may not have been controlled.

This study also proposed VAT threshold values to predict the onset of metabolic syndrome according to the IDF definition, a valuable element in interpreting the results obtained.

Also, the lockdown periods during the COVID-19 pandemic years may have impacted sedentary behaviour and physical activity in some participants.

## Conclusions

This study provides reference values for VAT measured using GE Healthcare Lunar iDXA in an urban Algerian adult population, highlights its importance in assessing cardiometabolic risk, and proposes VAT threshold values to predict metabolic syndrome. The practical implications of these findings are significant, as they provide a non-invasive and reliable method for identifying individuals at higher cardiometabolic risk.

## Supporting information

S1 Fig Normality curve of VAT mass. VAT mass does not follow a normal distribution (p < 0.001 according to the Shapiro-Wilk method).
(PDF)

S1 Table Other general characteristics of the study population. (PDF)

S2 Table Visceral Adipose Tissue values percentiles by gender. (PDF)

S3 Table Visceral Adipose Tissue mass by gender, age groups and health status. (PDF)

S4 Table Others suggested threshold values of VAT derived from Youden’s index for cardiometabolic risk factors. (PDF)
