# Supplementary material for: DXA-derived visceral adipose tissue reference values and metabolic syndrome risk threshold in an Algerian adult population
Source: PLoS One. 2025 Sep 9;20(9):e0331867. doi: 10.1371/journal.pone.0331867 (PMC12419631; doi:10.1371/journal.pone.0331867)

S1 Figure. Normality curve of VAT mass. VAT mass does not follow a normal distribution ($p < 0.001$ according to the Shapiro-Wilk method)

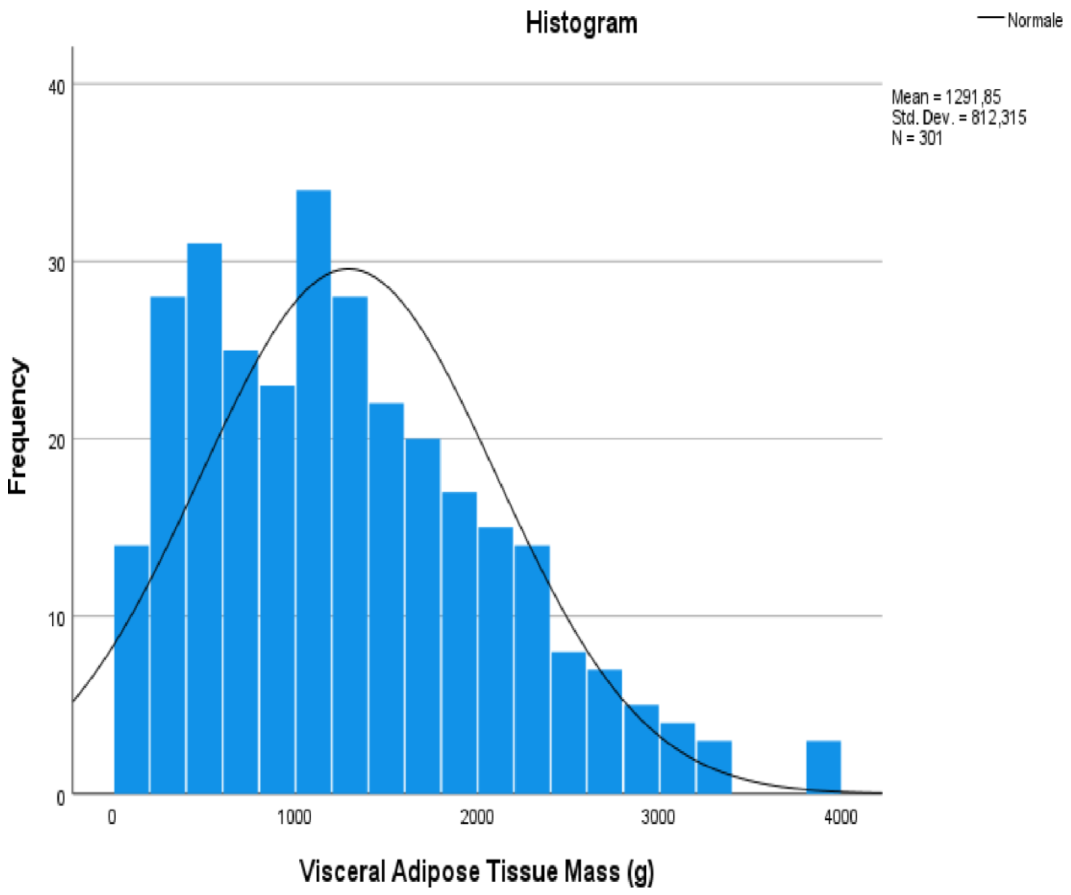

Supplement: S1 Fig — VAT mass does not follow a normal distribution (p < 0.001 according to the Shapiro-Wilk method). (PDF) [file pone.0331867.s001.pdf]
