# Supplementary material for: DXA-derived visceral adipose tissue reference values and metabolic syndrome risk threshold in an Algerian adult population
Source: PLoS One. 2025 Sep 9;20(9):e0331867. doi: 10.1371/journal.pone.0331867 (PMC12419631; doi:10.1371/journal.pone.0331867)
Supplement: S1 Table — (PDF) [file pone.0331867.s002.pdf]

S1 Table. Other general characteristics of the study population

	All (N = 301)	Men (N = 147)	Women (N = 153)	P value men vs. women
**Municipality of residence**				
Tlemcen	208 (69.1%)	101 (68.7%)	107 (69.5%)	0.350
Mansourah	52 (17.3%)	24 (16.3%)	28 (18.2%)	
Chetouane	16 (5.3%)	6 (4.1%)	10 (6.5%)	
Others	25 (8.3%)	16 (10.9%)	9 (5.8%)	
**Marital status**				
Married	214 (71.1%)	112 (76.2%)	102 (66.2%)	< 0.001*
Single	51 (16.9%)	33 (22.4%)	18 (11.7%)	
Widowed	26 (8.6%)	0 (0)	26 (16.9%)	
Divorced	10 (3.3%)	2 (1.4%)	8 (5.2%)	
**Educational level**				
None	16 (5.3%)	2 (1.4%)	14 (9.1%)	0.005*
Primary	29 (9.6%)	9 (6.1%)	20 (13%)	
Lower secondary	41 (13.6%)	22 (15%)	19 (12.3%)	
Upper secondary	81 (26.9%)	46 (31.3%)	35 (22.7%)	
Higher	134 (44.5%)	68 (46.3%)	66 (42.9%)	
**Profession or status**				
Permanent	149 (49.5%)	92 (62.6%)	57 (37%)	< 0.001*
Temporary	10 (3.3%)	5 (3.4%)	5 (3.2%)	
Unemployment	77 (25.6%)	8 (5.4%)	69 (44.8%)	
Retirement	39 (13%)	20 (13.6%)	19 (12.3%)	
Student	26 (8.6%)	22 (15%)	4 (2.6%)	
**Social insurance**				
Insured	272 (90.4%)	132 (89.8%)	140 (90.9%)	0.744
Uninsured	29 (9.6%)	15 (10.2%)	14 (9.1%)	
**Monthly Household income**				
≤ 20 000 Dinars	11 (3.7%)	6 (4.1%)	5 (3.2%)	0.507
20 000 – 40 000 Dinars	36 (12%)	15 (10.2%)	21 (13.6%)	
40 000 – 100 000 Dinars	152 (50.4%)	80 (54.4%)	72 (46.8%)	
> 100 000 Dinars	102 (33.9%)	46 (31.3%)	56 (36.4%)	

*: significant difference between men and women with p-value < 0.05
