# Supplementary material for: DXA-derived visceral adipose tissue reference values and metabolic syndrome risk threshold in an Algerian adult population
Source: PLoS One. 2025 Sep 9;20(9):e0331867. doi: 10.1371/journal.pone.0331867 (PMC12419631; doi:10.1371/journal.pone.0331867)
Supplement: S2 Table — (PDF) [file pone.0331867.s003.pdf]

S2 Table. Visceral Adipose Tissue values percentiles by gender

	Men (N = 147)	Women (N = 153)
**VAT mass** (g)		
5e percentile	179.4	235.7
10e percentile	318.0	304.5
25e percentile	690.0	585.7
50e percentile	1364.0	1060.0
75e percentile	2049.0	1590.0
90e percentile	2738.6	2173.0
95e percentile	3117.6	2484.5
**VAT volume** (cm^3)		
5e percentile	189.8	249.7
10e percentile	337.0	323.0
25e percentile	731.0	620.7
50e percentile	1446.0	1123.5
75e percentile	2172.0	1685.5
90e percentile	2902.2	2303.5
95e percentile	3304.8	2633.2
**VAT area** (cm^2)		
5e percentile	21.4	29.7
10e percentile	36.6	38.0
25e percentile	79.0	72.7
50e percentile	154.0	131.5
75e percentile	235.0	192.7
90e percentile	292.8	256.5
95e percentile	347.2	314.0

VAT: Visceral Adipose Tissue.
