# Supplementary material for: DXA-derived visceral adipose tissue reference values and metabolic syndrome risk threshold in an Algerian adult population
Source: PLoS One. 2025 Sep 9;20(9):e0331867. doi: 10.1371/journal.pone.0331867 (PMC12419631; doi:10.1371/journal.pone.0331867)
Supplement: S3 Table — (PDF) [file pone.0331867.s004.pdf]

S3 Table. Visceral Adipose Tissue mass by gender, age groups and health status

	Men		
	**Healthy** (N = 66)	**Unhealthy** (N = 81)	**P value healthy vs. unhealthy**
**VAT mass** (g)			
All	800.5 (337.0-1348.0)	1872.0 (1282.5-2350.5)	< 0.001*
18 to ≤ 30 years	338.0 (173.5-532.5)	-	-
> 30 to ≤ 40 years	1008.5 (655.2-1245.2)	2517.0 (1240.5-3076.7)	< 0.001*
> 40 to ≤ 50 years	1413.0 (996.5-2015.5)	1786.0 (1374.0-2372.0)	0.151
>50 to ≤ 60 years	1278.0 (596.2-1525.75)	1773.0 (1128.5-2166.0)	0.035*
> 60 to ≤ 70 years	2070.0 (1348.0-2792.0)	2081.0 (1344.0-2482.0)	0.933
> 70 to 88 years	-	1845.0 (1377.2-2078.0)	-
	**Women**		
	**Healthy** (N = 58)	**Unhealthy** (N = 96)	**P value healthy vs. unhealthy**
**VAT mass** (g)			
All	642.5 (359.0-1046.0)	1309.5 (838.7-1884.5)	< 0.001*
18 to ≤ 30 years	567.0 (361.5-705.0)	-	-
> 30 to ≤ 40 years	595.0 (409.0-879.0)	1113.5 (603.5-1287.5)	0.064
> 40 to ≤ 50 years	808.0 (395.5-1038.5)	1459.5 (784.5-1692.0)	0.004*
>50 to ≤ 60 years	341.0 (279.7-925.5)	1384.0 (990.5-1968.0)	0.003*
> 60 to ≤ 70 years	1150.5 (304.5-1428.0)	1198.0 (843.5-1913.5)	0.142
> 70 to 88 years	-	1293.0 (855.0-2329.5)	-

VAT: Visceral Adipose Tissue. VAT mass is represented in median (25th-75th percentile). An individual is considered healthy if they're free from conditions like hypertension, type 2 diabetes, dyslipidemia, metabolic syndrome, cardiovascular events, sleep apnea syndrome, and hepatic steatosis. *: significant difference with p-value < 0.05.
