# Supplementary material for: DXA-derived visceral adipose tissue reference values and metabolic syndrome risk threshold in an Algerian adult population
Source: PLoS One. 2025 Sep 9;20(9):e0331867. doi: 10.1371/journal.pone.0331867 (PMC12419631; doi:10.1371/journal.pone.0331867)
Supplement: S4 Table — (PDF) [file pone.0331867.s005.pdf]

S4 Table. Others suggested threshold values of VAT derived from Youden's index for cardiometabolic risk factors

	AUC (95% CI)	VAT thresholds values		Youden's Index	Sensitivity (%)	Specificity (%)
		**Mass** (g)	**Volume** (cm^3)			
**Hypertension**						
Men	0.710 (0.622–0.797)	≥ 1480	≥ 1569,5	0.416	72.7	68.9
Women	0.733 (0.651–0.814)	≥ 1534,5	≥ 1626	0.384	48.4	90
**Type 2 Diabetes**						
Men	0.738 (0.645–0.830)	≥ 1499	≥ 1589	0.475	79.4	68.1
Women	0.712 (0.623–0.802)	≥ 1082,5	≥ 1147,5	0.339	73.3	60.6
**Dyslipidemia**						
Men	0.796 (0.718–0.874)	≥ 1499	≥ 1589	0.486	78.9	69.7
Women	0.651 (0.553–0.748)	≥ 1106	≥ 1172,5	0.283	69.2	59.1
**Insulin resistance by HOMA2-IR**						
Men	0.783 (0.704–0.863)	≥ 1220	≥ 1293,5	0.527	76.8	75.9
Women	0.756 (0.672–0.840)	≥ 1106	≥ 1172,5	0.412	62	79.2
**Hepatic steatosis**						
Men	0.834 (0.724–0.943)	≥ 2350	≥ 2491,5	0.583	70	88.3
Women	0.687 (0.447–0.926)	≥ 1675	≥ 1775	0.399	60	79.9
**Sleep apnea syndrome**						
Men	0.746 (0.554–0.939)	≥ 1993,5	≥ 2113	0.546	80	74.6
Women	0.805 (0.683–0.927)	≥ 1036	≥ 1098	0.510	100	51

AUC: Area Under the Curve, VAT: Visceral adipose tissue,
